# p53 pathway determines the cellular response to alcohol-induced DNA damage in MCF-7 breast cancer cells

**DOI:** 10.1371/journal.pone.0175121

**Published:** 2017-04-03

**Authors:** Ming Zhao, Erin W. Howard, Zhiying Guo, Amanda B. Parris, Xiaohe Yang

**Affiliations:** Department of Biological and Biomedical Sciences, Julius L. Chambers Biomedical/Biotechnology Research Institute (BBRI), North Carolina Central University, Kannapolis, North Carolina; Roswell Park Cancer Institute, UNITED STATES

## Abstract

Alcohol consumption is associated with increased breast cancer risk; however, the underlying mechanisms that contribute to mammary tumor initiation and progression are unclear. Alcohol is known to induce oxidative stress and DNA damage; likewise, p53 is a critical modulator of the DNA repair pathway and ensures genomic integrity. p53 mutations are frequently detected in breast and other tumors. The impact of alcohol on p53 is recognized, yet the role of p53 in alcohol-induced mammary carcinogenesis remains poorly defined. In our study, we measured alcohol-mediated oxidative DNA damage in MCF-7 cells using 8-OHdG and p-H2AX foci formation assays. p53 activity and target gene expression after alcohol exposure were determined using p53 luciferase reporter assay, qPCR, and Western blotting. A mechanistic study delineating the role of p53 in DNA damage response and cell cycle arrest was based on isogenic MCF-7 cells stably transfected with control (MCF-7/Con) or p53-targeting siRNA (MCF-7/sip53), and MCF-7 cells that were pretreated with Nutlin-3 (Mdm2 inhibitor) to stabilize p53. Alcohol treatment resulted in significant DNA damage in MCF-7 cells, as indicated by increased levels of 8-OHdG and p-H2AX foci number. A p53-dependent signaling cascade was stimulated by alcohol-induced DNA damage. Moderate to high concentrations of alcohol (0.1–0.8% v/v) induced p53 activation, as indicated by increased p53 phosphorylation, reporter gene activity, and p21/Bax gene expression, which led to G0/G1 cell cycle arrest. Importantly, compared to MCF-7/Con cells, alcohol-induced DNA damage was significantly enhanced, while alcohol-induced p21/Bax expression and cell cycle arrest were attenuated in MCF-7/sip53 cells. In contrast, inhibition of p53 degradation via Nutlin-3 reinforced G0/G1 cell cycle arrest in MCF-7 control cells. Our study suggests that functional p53 plays a critical role in cellular responses to alcohol-induced DNA damage, which protects the cells from DNA damage associated with breast cancer risk.

## Introduction

Data from epidemiological studies support that alcohol consumption increases breast cancer risk, especially in cases of cumulative alcohol intake throughout adult life, premenopausal women, and combined exposure to alcohol and tobacco [1–7]. Despite the significant link between alcohol consumption and increased breast cancer risk from clinical data, the molecular mechanisms behind alcohol-associated carcinogenesis are not fully understood. Available data suggest that alcohol-associated breast carcinogenesis activates several pathways involving oxidative stress, endocrine disruption, and epigenetic alterations [8–10]. However, critical molecules and signaling mechanisms that mediate specific cellular responses remain to be defined. Therefore, understanding the molecular mechanism of alcohol-associated breast cancer risk is of pivotal importance in breast cancer prevention and management.

Increasing evidence, including our previous findings, suggests that oxidative stress, resulting from alcohol metabolism, is a primary culprit for the increased risk and progression of alcohol-associated breast cancer [10, 11]. Alcohol is metabolized mostly via oxidation to acetaldehyde by alcohol dehydrogenase (ADH) and microsomal cytochrome P450 2E1 (CYP2E1) [12, 13]. The resulting acetaldehyde is further oxidized by acetaldehyde dehydrogenase (ALDH) to acetate. This metabolic process is accompanied by the generation of reactive oxygen species (ROS) and the induction of oxidative stress [12, 13]. Alcohol-associated oxidative stress can induce a variety of alterations/damage to DNA, including DNA adducts, DNA strand breaks, and interstrand DNA crosslinks [14–17]. The formation of consequential oxidative DNA damage and adducts is considered an essential initiating event in alcohol-related cancer development [14]. Consistently, reports from *in vivo* data also demonstrate that alcohol consumption promotes oxidative stress and produces ultrastructural chromatin alterations in mammary epithelial cells [10]; thus, supporting the role of alcohol-induced genetic instability in breast carcinogenesis. In turn, the DNA damaging effects of oxidative stress leads to the activation of the p53 pathway [18].

p53 is a well-established tumor suppressor that plays a vital role in genomic homeostasis, cell cycle regulation, and apoptosis induction in response to various cellular stresses, especially DNA damage [19–22]. Previous studies reveal that the cellular response to oxidative stress and DNA damage recruits ataxia telangiectasia mutated (ATM)/ATM and Rad3 related (ATR) to the damaged sites [23, 24]. Sequentially, ATM/ATR kinase activity, Chk2 phosphorylation/activation, and Mdm2 inhibition work together to stabilize and activate p53 [21, 24, 25]. p53 exerts its action through transcriptional regulation of p21, Bax, and other key factors involved in DNA damage repair, cell cycle arrest, and apoptosis. As such, p53 mutations have been detected in the majority of human cancers and are associated with poor prognosis [26–28]. Importantly, the frequency of p53 gene mutations varies between breast cancer subtypes, which can be up to 70–80% in basal-like or ErbB2-overexpressing breast cancers [29, 30]. Nevertheless, studies on p53 in alcohol-associated carcinogenesis remain sporadic. It was reported that p53 mutations increased in tumors from alcohol drinkers as compared to tumors from patients who have never consumed alcohol [26–28]. It also appears that combined alcohol and tobacco exposure may amplify the frequency of p53 mutations, as in a study based on tumors from patients with non-small cell lung cancer [27]. This is further supported by the report that alcohol exposure enhanced UV radiation-induced p53 mutations in skin tumors [31]. While the connection between p53 function and alcohol-associated carcinogenesis has been recognized, the specific role of p53 in alcohol-induced cellular responses and breast cancer has not been studied in depth. In particular, DNA damage-induced activation of p53 family members, such as p63 and p73, may also regulate p53 target genes. Indeed, several studies report that p21 induction can occur independent of p53 by other regulatory proteins [32–34]. For instance, BRCA1 can modulate p21 through p53-dependent and -independent mechanisms [34]. Although these alternative pathways exist, p53 is still the most efficient regulator of p21 and the cell cycle, making functional p53 expression and activation of utmost importance in response to cellular stresses. Given the complexity of the p53 regulatory network, it is crucial to differentiate p53-dependent and -independent regulation in alcohol-induced cellular responses in breast cancer cells. To this end, the development of p53-specific models for alcohol studies will facilitate our understanding of this critical issue.

In this report, based on paired breast cancer cell lines with specific p53 knockdown, we investigated the particular role of p53 in alcohol-induced DNA damage and cell cycle arrest. The MCF-7 breast cancer cell line harbors endogenous wild-type p53 expression and is often used as a cellular model for detecting alcohol-induced carcinogenesis [35–37]. We demonstrated that alcohol exposure triggers a p53-dependent signaling cascade and cell cycle arrest, while p53 knockdown influenced alcohol-induced activities as indicated by attenuated cell cycle arrest and intensified DNA damage. In contrast, treatment with Nutlin-3, a p53 stabilizer, enhanced G1 cell cycle arrest in wild-type MCF-7 cells. Taken together, we highlight the importance of p53 signaling in maintaining genomic integrity *in vitro*, alongside the potential role of p53 in preventing alcohol-related breast cancer initiation and progression.

## Materials and methods

### Reagents and antibodies

Absolute (200 proof) ethanol was purchased from Fisher Scientific (Waltham, MA). Nutlin-3 was purchased from Sigma-Aldrich (St Louis, MO). The following primary antibodies were purchased from Santa Cruz Biotechnology (Santa Cruz, CA): p21 (C-19), Bax (N-20), BRCA1 (D-9), and β-actin (C4); and p53, p-p53 (Ser15), p-p53 (Ser20), H2AX, p-H2AX (Ser139), ATM, p-ATM (Ser1981), Chk2, p-Chk2 (Thr68), p-BRCA1 (Ser1524), PARP, and cleaved PARP primary antibodies were purchased from Cell Signaling Technology (Danvers, MA).

#### Cell culture

MCF-7 cells (ATCC; Manassas, VA) were maintained in DMEM/F-12 medium (Gibco; Gaithersburg, MD) supplemented with 10% fetal bovine serum (FBS), 100 U/ml penicillin, and 100 μg/ml streptomycin at 37°C in a humidified atmosphere of 5% CO_2_. MCF-7/sip53, a stable isogenic subline of the MCF-7 cell line with p53 knocked down, was established in our lab as described previously [38]. For alcohol exposure, cells were treated with alcohol in fresh medium 24 hours after initial seeding. Because alcohol evaporation can affect treatment concentrations, cell culture dishes were placed within a larger dish containing filter paper immersed in the same alcohol concentration as in the cell culture medium. For Nutlin-3 treatments, Nutlin-3 was dissolved in DMSO and diluted in complete medium 1 hour prior to alcohol treatments. In the control groups, equal volumes of PBS were added in the culture medium.

#### DNA oxidative damage assay

Alcohol-induced DNA damage was determined using a DNA Oxidative Damage ELISA kit (Cayman Chemical; Ann Arbor, MI) according to the manufacturer’s instructions. Briefly, genomic DNA was isolated using the Wizard genomic DNA purification kit (Promega; Madison, WI) and then digested with nuclease P1 and alkaline phosphatase to yield free deoxy-guanosine (dG). After boiling the samples for 10 minutes and centrifugation for 5 minutes at 6000 g, the supernatant was collected for the 8-hydroxy-2-deoxy-guanosine (8-OHdG) ELISA assay. 8-OHdG antibody and sample DNA were added to a 96-well plate pre-coated with 8-OHdG and incubated at 4°C overnight. The plate was later incubated with horseradish peroxidase-conjugated secondary antibody for 1 hour at room temperature, followed by a 15 minute substrate reaction with 3,3′, 5,5′-tetramethylbenzidine. The reaction was terminated by the addition of phosphoric acid and absorbance was measured at 405 nm. All assays were performed in triplicate and the results are expressed as relative fold changes in the alcohol-treated samples as compared to the control samples.

#### Immnunofluorescence

Cells cultured on coverslips were washed twice in PBS and fixed in 4% paraformaldehyde for 15 minutes. The cells were then washed 3 times with PBS and permeabilized with 0.5% triton X-100 for 10 minutes, followed by blocking incubation in 1% BSA/PBS solution for 1 hour. The slides were stained overnight at 4°C with primary anti-p-H2AX monoclonal antibody (1:100 dilution in 1% BSA/PBS). Then, after washing with PBS 3 times, the cells were incubated with secondary goat anti-mouse Alexa-488-conjugated IgG (1:1000 dilution in 1% BSA/PBS) for 1 hour at room temperature. After washing with PBS 3 times, samples were immersed in 2 ng/ml DAPI/PBS solution for 15 minutes for nuclei counterstaining. The coverslips were mounted on glass slides with VECTASHIELD Mounting Media (Vector Laboratories; Burlingame, CA). Images of p-H2AX and DAPI stained samples were obtained using a confocal microscope LSM880 with AxioObserver. Optical sections (1.2 μm) through the cell nuclei were imaged. The number of DNA damage foci with p-H2AX-positive staining was quantified in three independent experiments. At least 100 cell nuclei in each group were counted for statistical analysis.

#### Western blot

The cells were lysed in Laemmli sample buffer (Bio-Rad; Hercules, CA), and the protein concentrations were determined with a BCA assay (Fisher Scientific). Equal amounts of protein (50 μg) from cell lysates were resolved by SDS-PAGE and transferred to nitrocellulose membranes (Millipore; Billerica, MA). The membranes were blocked in TBST with 5% non-fat milk and then incubated with the primary antibodies at appropriate dilutions at 4°C overnight. Membranes were washed with TBST solution, followed by incubation with horseradish peroxidase-linked secondary antibody (1:3000) in TBST with 5% non-fat milk for 1 hour at room temperature. Finally, chemiluminescence was detected using the Super Signal West Dura Detection System (Fisher Scientific) and the protein bands were visualized with Cell Biosciences FluorChemE imager (Santa Clara, CA).

#### Luciferase reporter assay

MCF-7 cells were plated at 1×10^5^ cells/well in 12-well plates. The next day, the cells were transfected with 0.5 μg of the reporter plasmid in each well using X-tremeGENE 9 DNA transfection reagent (Roche; Indianapolis, IN), according to the protocol provided by the manufacturer, for 48 hours. After 48 hours of transfection, the MCF-7/p53-luc cells were then treated with various doses of alcohol (0, 0.2%, or 0.4% v/v) for 2 hours. After lysate normalization with BCA assays, luciferase activity in individual samples was measured using the Luciferase Assay system (Promega) and the Modulus Single Tube Luminometer (Turner BiosyStems; Sunnyvale, CA).

#### Quantitative PCR

Quantitative PCR (qPCR) was performed as previously described [39]. Briefly, total RNA was isolated using TRIzol Reagent (Invitrogen; Carlsbad, CA) and reverse transcribed to cDNA with iScript cDNA Synthesis kit (Bio-Rad). qPCR assays were carried out in triplicate with Bio-Rad CFX96 using SYBR green. GAPDH was used as an internal control and the relative mRNA levels were calculated using the comparative-Ct method (^ΔΔ^Ct method).

#### Cell cycle analysis

Cells were harvested by trypsin digestion and washed with cold PBS. Then, the cells were fixed with ice cold 70% ethanol drop-wise and stored at -20°C overnight. The fixed cells were collected by centrifugation at 2000 rpm for 5 minutes and resuspended in PBS containing 0.2% triton X-100, 500 μg/ml RNase A, and 33 μg/ml propidium iodide. After incubation for 45 minutes at 37°C, samples were subjected to FACS analysis. The data were analyzed by ModFit software.

#### Statistical analysis

Data presented as means ± S.E. were analyzed by GraphPad Prism Version 5 (GraphPad Software Inc.; San Diego, CA). Student's paired t-tests were used to measure statistical differences between groups and p<0.05 was considered to be statistically significant.

## Results

### Alcohol induces oxidative DNA damage in MCF-7 cells

**Fig 1:**
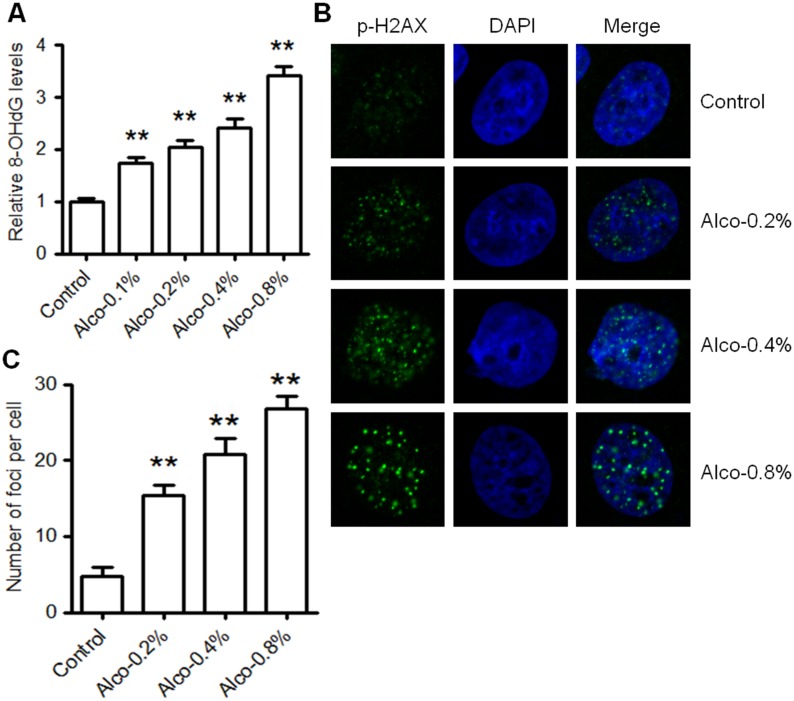
Alcohol induces DNA damage in MCF-7 cells. **A)** MCF-7 cells were treated with alcohol (0, 0.1%, 0.2%, 0.4%, or 0.8% v/v) for 2 hours. Then DNA damage was quantified using an 8-OHdG ELISA assay and was performed in triplicate. The graph represents the relative levels of DNA damage (8-OHdG) as compared to the control. Values are presented as the mean ± standard error of the mean (S.E.) (**p<0.01). **B)** MCF-7 cells were cultured on glass coverslips and treated with various doses of alcohol (0, 0.2%, 0.4%, or 0.8% v/v) for 2 hours. After incubation in alcohol, cells were fixed and stained with p-H2AX primary and Alexa-488-conjugated secondary antibodies. Then, nuclei were counterstained with DAPI. Representative images are shown for each control and treatment group with p-H2AX foci indicating DSBs in the nuclei. **C)** The graph depicts the mean number of foci per cell (± S.E.) in each group as shown in B (**p<0.01).

Previous studies have reported that alcohol exposure stimulates oxidative stress and produces DNA damage in cells [10, 14]. To examine the induction of oxidative DNA damage after alcohol treatment in MCF-7 cells, we assayed levels of 8-OHdG, one the most abundant oxidative products of cellular DNA. As shown in Fig 1A, oxidative DNA damage in MCF-7 cells was markedly enhanced by physiologically relevant and high concentrations of alcohol (0.1–0.8% v/v), as represented by a dose-dependent increase of relative 8-OHdG levels in the cellular DNA. Another indicator of DNA damage, specifically double-strand breaks (DSBs), is the rapid phosphorylation of H2AX at Ser 139 and the subsequent appearance of nuclear foci that contain proteins involved in DNA repair and checkpoint signaling. Thus, p-H2AX (also known as γ-H2AX) is a sensitive marker of DSBs [40]. With immunofluorescence staining, we found that alcohol induces p-H2AX foci formation in a dose-dependent manner in MCF-7 cells (Fig 1B and 1C). Our results demonstrate that alcohol exposure induces significant oxidative DNA damage in MCF-7 cells even at moderate/physiologically relevant concentrations of alcohol.

### Alcohol activates the DNA damage response pathway in MCF-7 cells

**Fig 2:**
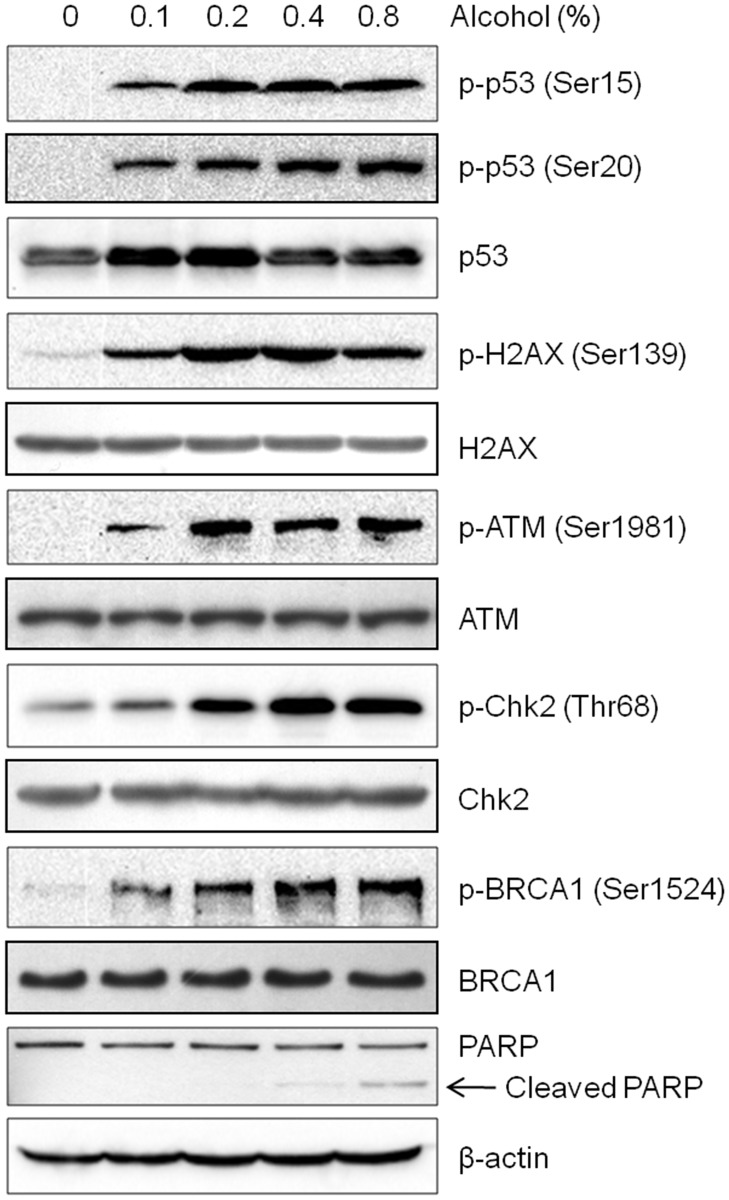
Alcohol activates the DNA damage/p53 pathway. MCF-7 cells were treated with alcohol (0, 0.1%, 0.2%, 0.4%, or 0.8% v/v) for 2 hours. Then cells were harvested and protein was extracted for Western blot analysis of the indicated markers.

Previous studies have shown that alcohol, as well as acetaldehyde, results in DNA damage and activates the Fanconi anemia-breast cancer (FA-BRCA) DNA damage response network [17, 41]. To confirm the ability of alcohol to trigger DNA damage response systems in MCF-7 cells, we examined the changes in protein expression of DNA damage markers in response to alcohol treatment. As shown in Fig 2, moderate to high concentrations of alcohol (0.1–0.8% v/v) caused significant increases in the phosphorylation of ATM, Chk2, p53, BRCA1, and H2AX, all of which are involved in DSB response signaling, in a dose-dependent manner [21, 24, 42]. In contrast, total/unphosphorylated protein expression of ATM, Chk2, BRCA1, and H2AX remained unchanged after alcohol exposure. It is important to highlight that the increase of phosphorylated p53 (Ser15 and Ser20) and total p53 implies that the p53 pathway, the most essential pathway protecting genomic integrity, was activated in response to alcohol-induced DNA damage in MCF-7 cells. Additionally, we found that high concentrations of alcohol (0.4% and 0.8% v/v) also induced apoptosis in MCF-7 cells, as indicated by PARP cleavage. Together these data demonstrate the complex DNA damage response network that is activated by alcohol exposure in MCF-7 breast cancer cells.

### Alcohol activates the p53 pathway and induces cell cycle arrest in MCF-7 cells

**Fig 3:**
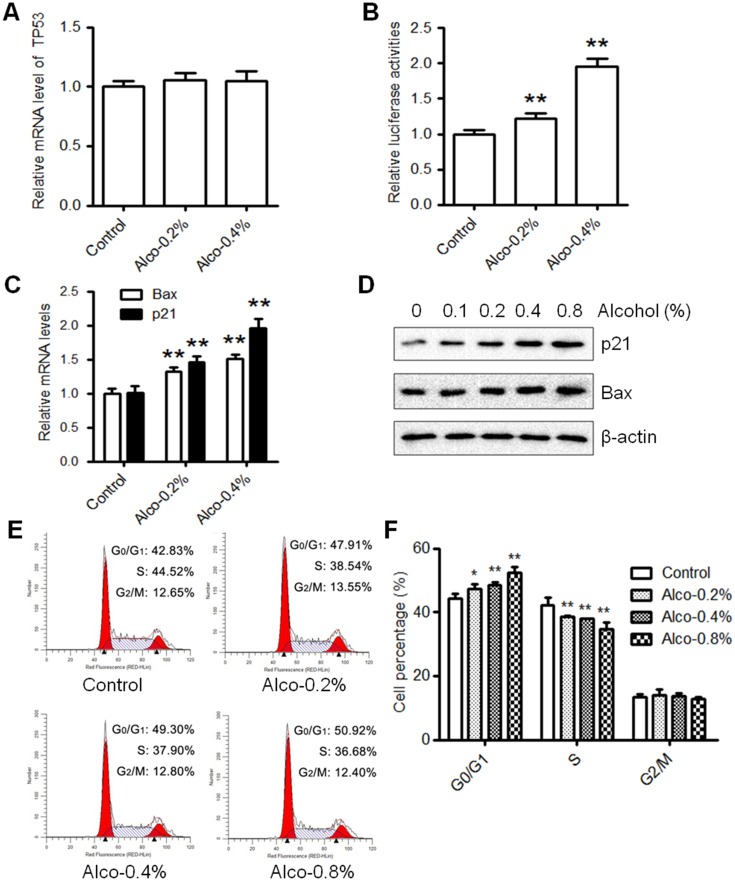
Alcohol increases p53 transcriptional activity and induces cell cycle arrest. **A)** Relative mRNA levels of *TP53* are shown from MCF-7 cells treated with alcohol (0, 0.2%, or 0.4% v/v) for 6 hours. Values are presented as the mean ± S.E. **B)** MCF-7 cells transiently transfected with the p53 luciferase reporter plasmid (MCF-7/p53-luc cells) were treated with alcohol (0, 0.2%, or 0.4% v/v) for 2 hours. Then, the cell lysates were prepared for reporter assays. The luciferase activity relative to the control for each sample is shown. Values are presented as the mean ± S.E. (**p<0.01). **C)** MCF-7 cells were treated with various doses of alcohol (0, 0.1%, 0.2%, or 0.4% v/v) for 6 hours. mRNA levels of p21 and Bax were measured using qPCR. Relative fold changes for the alcohol-treated samples are displayed as compared to the respective control. Values are presented as the mean ± S.E. (**p<0.01). **D)** MCF-7 cells were treated with alcohol (0, 0.1%, 0.2%, 0.4%, or 0.8% v/v) for 6 hours and then harvested for protein expression. p21 and Bax protein levels were determined by Western blot analysis. **E)** MCF-7 cells were exposed to alcohol (0, 0.2%, 0.4%, or 0.8% v/v) for 24 hours before cells were fixed and prepared for FACS analysis as described in the *Materials and Methods*. **F)** The graph depicts the average percentage of cells (± S.E.) in G0/G1, S, and G2/M phase (*p<0.05; **p<0.01 as compared to the control) from three replicate experiments.

To further investigate the effect of alcohol on the activation of the p53 pathway, we first detected the effect of alcohol on *TP53* (p53 gene) mRNA levels. As shown in Fig 3A, we found that alcohol exposure (0.2% or 0.4% v/v) did not significantly modify the relative expression of *TP53* as compared to control-treated MCF-7 cells. Next, we measured p53 transcriptional activity by transfecting MCF-7 cells with a p53 luciferase reporter plasmid. The luciferase activity after alcohol (0.2–0.4% v/v) exposure was evaluated and our results show that alcohol increased p53 luciferase reporter activity dose-dependently (Fig 3B). Similarly, the mRNA levels of downstream p53 target genes, p21 and Bax, were upregulated by alcohol exposure (Fig 3C), indicating that the transcriptional activity of p53 was also increased. Based on these data, the increased p53 protein levels (Fig 2) and p53 transcriptional activity (Fig 3B and 3C) are likely due to enhanced protein stability after phosphorylation/activation. Consistently, the protein levels of p21 and Bax were amplified after alcohol exposure as well (Fig 3D). Alongside the induction of p21, an established cyclin-dependent kinase (Cdk) inhibitor that blocks the G1 to S phase transition of the cell cycle, alcohol significantly induced G0/G1 cell cycle arrest and reduced the percentage of proliferating cells in S phase in a dose-dependent manner (Fig 3E and 3F). These results demonstrate that alcohol can activate the p53 signaling cascade and induce cell cycle arrest in MCF-7 cells under the given conditions.

### Knockdown of p53 alleviates alcohol-induced cell cycle arrest in MCF-7 cells

**Fig 4:**
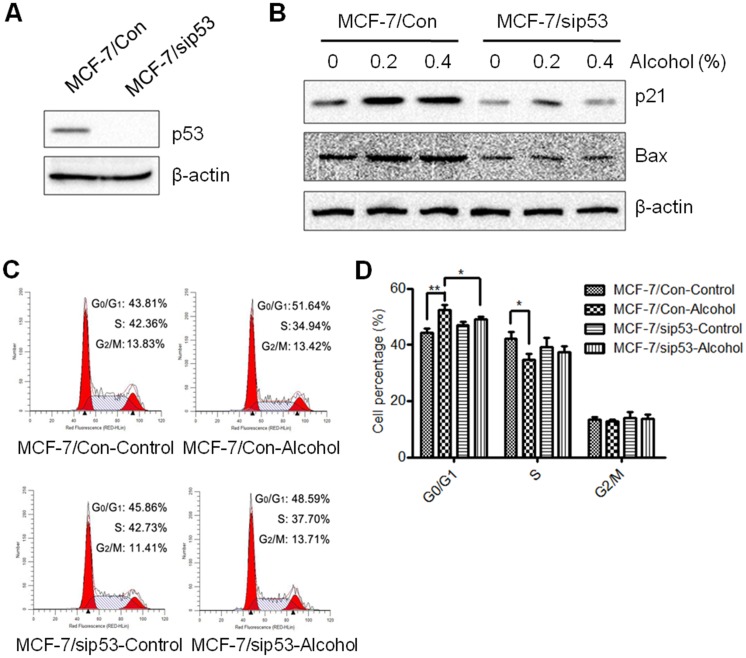
p53 knockdown alleviates alcohol-induced cell cycle arrest. **A)** p53 was knocked down in MCF-7 cells that were stably transfected with p53 siRNA as verified by Western blot analysis. **B)** MCF-7 control (MCF-7/Con) and MCF-7 p53-knockdown (MCF-7/sip53) cells were treated with various doses of alcohol (0, 0.2%, or 0.4% v/v) for 2 hours. Protein expression of p53 targets, p21 and Bax, were detected by Western blot analysis. **C)** MCF-7/Con and MCF-7/sip53 cells were treated with alcohol (0 or 0.8% v/v) for 24 hours. Then, the cells were prepared for FACS analysis. **D)** The graph depicts the average percentage of cells (± S.E.) in G0/G1, S, and G2/M phase (*p<0.05; **p<0.01) from three replicate experiments.

In order to identify the specific role of p53 in alcohol-induced cellular responses, we knocked down the expression of p53 in MCF-7 cells using siRNA, as verified in Fig 4A. In the isogenic, stably transfected MCF-7/sip53 cell line that we generated, we looked at the protein levels of p53 targets (i.e. p21 and Bax) after alcohol exposure (0.2–0.4% v/v). As shown in Fig 4B, baseline and alcohol-induced expression of p21 and Bax were blocked in p53 knockdown cells, as compared to control cells. Simultaneously, alcohol-induced cell cycle arrest was alleviated in p53 knockdown cells (Fig 4C and 4D). Our data suggest that p53 and its targets play a crucial role in cell cycle regulation that allows for the repair of alcohol-induced oxidative DNA damage in MCF-7 cells.

### p53 knockdown renders MCF-7 cells more sensitive to alcohol-induced DNA damage

**Fig 5:**
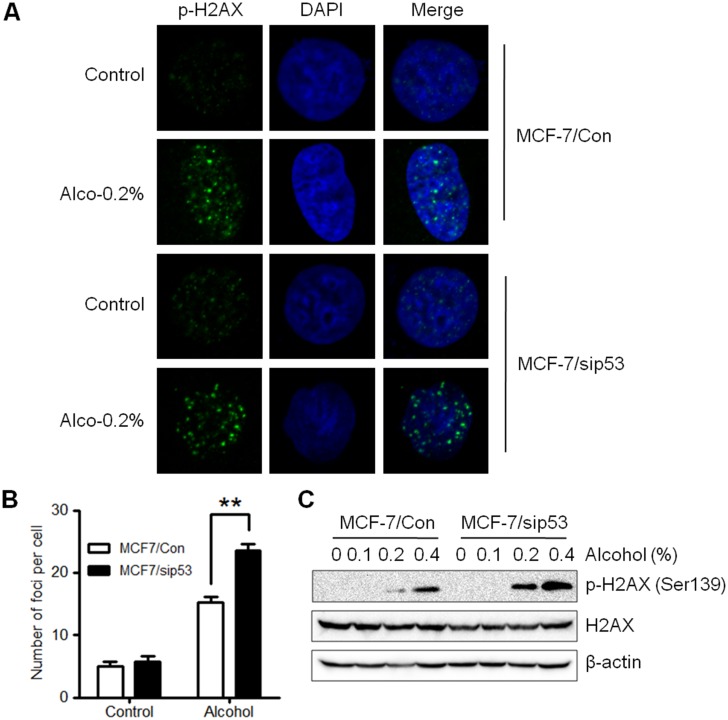
p53 knockdown increases alcohol-induced DNA damage. **A)** MCF-7/Con and MCF-7/sip53 cells were cultured on glass coverslips and exposed to various doses of alcohol (0 or 0.2% v/v) for 2 hours. Then, the cells were fixed and stained with p-H2AX primary and Alexa-488-conjugated secondary antibodies. The nuclei were counterstained with DAPI. Representative images are shown for each control and treatment group with p-H2AX foci formation in the nuclei. **B)** The graph depicts the mean number of foci per cell (± S.E.) in each group as shown in A (**p<0.01). **C)** MCF-7/Con and MCF-7/sip53 cells were exposed to various doses of alcohol (0, 0.1%, 0.2%, or 0.4% v/v) for 2 hours before cells were harvested and prepped for Western blot analysis. p-H2AX, H2AX, and β-actin protein levels are shown.

To further evaluate the importance of p53 in MCF-7 cells, we examined the role of p53 in alcohol-induced oxidative DNA damage. p-H2AX foci formation and protein levels were measured by immunofluorescence and Western blot analysis, respectively, in MCF-7 p53 control and knockdown cells. Our results show that alcohol induced a more distinct increase of p-H2AX foci in p53 knockdown cells (Fig 5A and 5B) as compared to the MCF-7 control cells. The same pattern was displayed in the Western blot with more p-H2AX protein expression in alcohol-treated p53 knockdown cells (Fig 5C). Therefore, these results support that p53 is involved in maintaining DNA integrity in MCF-7 cells.

### p53 stabilization increases alcohol-induced cell cycle arrest and DNA damage in MCF-7 cells

**Fig 6:**
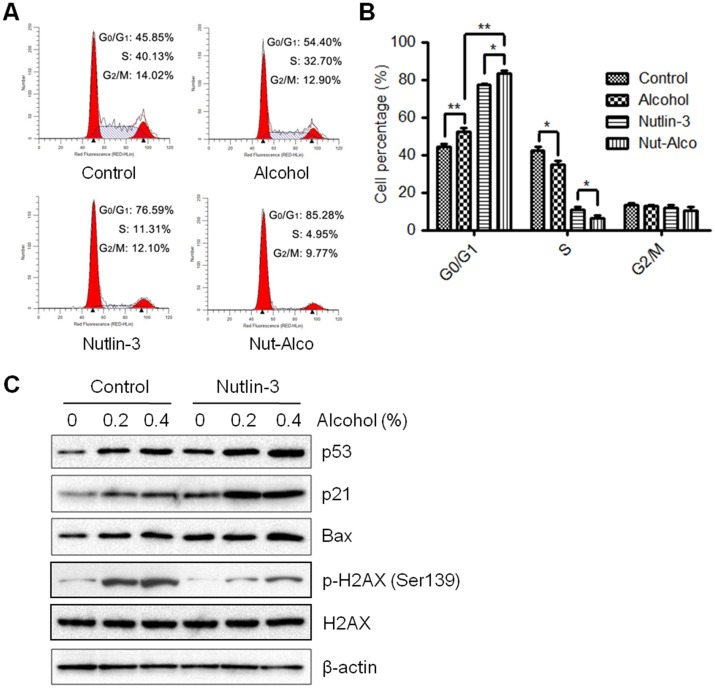
Nutlin-3 (an Mdm2 inhibitor) increases G0/G1 arrest induced by alcohol exposure. **A)** MCF-7 cells that were pretreated with 0 or 1 μM of Nutlin-3 for 1 hour were exposed to alcohol (0 or 0.8% v/v) for 24 hours. Cell cycle arrest was measured by FACS analysis. **B)** The graph depicts the average percentage of cells (± S.E.) in G0/G1, S, and G2/M phase (*p<0.05; **p<0.01) from three replicate experiments. **C)** p53, p21, Bax, p-H2AX, and H2AX protein expression were determined by Western blot analysis of cells that were pretreated with 0 or 1 μM of Nutlin-3 for 1 hour and then treated with alcohol (0, 0.2%, or 0.4% v/v) for 6 hours.

Nutlin-3, a small molecule Mdm2 inhibitor, binds preferentially to the p53-binding pocket of Mdm2, leading to p53 stabilization and enhanced p53 functions. Therefore, we used Nutlin-3 to enhance p53 expression to determine the impact of functional p53 on alcohol-induced cellular responses. We show that Nutlin-3 alone can induce cell cycle arrest at G0/G1 phase; however, the combination of Nutlin-3 and alcohol results in significantly amplified cell cycle arrest in MCF-7 cells (Fig 6A and 6B). Consistently, the basal and alcohol-induced protein levels of p53, as well as its targets p21 and Bax, are substantially increased after Nutlin-3 treatment (Fig 6C). We further demonstrated that Nutlin-3 pretreatment suppressed alcohol-induced DNA damage, as indicated by reduced H2AX phosphorylation as compared to the control cells. These results additionally confirm the role of p53 in alcohol-induced cell cycle arrest and DNA damage *in vitro*.

## Discussion

In our current study, we demonstrated the specific role of p53 in alcohol-induced cellular responses, which is connected to alcohol-induced oxidative DNA damage. In particular, alcohol exposure in cells with p53 knockdown significantly impaired the activation of p53 target genes and cell cycle arrest. We also demonstrated that p53 knockdown renders cells more sensitive to alcohol-induced DNA damage. These results provide direct evidence of p53-dependent regulation in alcohol-treated breast cancer cells. This study not only advanced our understanding of p53-regulated responses, but also provides a model system for further investigation of these responses in alcohol-associated breast cancer risk. Previously, the association between alcohol consumption and p53 mutation/deregulation in breast cancer etiology was mainly based on epidemiologic studies. In contrast to studies exploring the specific role of p53 in alcohol-induced pathogenesis in other cancers, such as hepatocellular carcinoma, similar models for breast cancer studies are missing [43]. To our knowledge, we are the first to use this *in vitro* model system to study the specific role of p53 in alcohol-associated breast cancer risk. This p53-specific paired cell line model will enable us to differentiate the function of p53 from cellular responses regulated by other factors/pathways.

MCF-7 cells are a luminal epithelial-like breast cancer cell line that is responsive to estrogen treatment [44, 45]. Since up to 80% of breast cancers are estrogen receptor-positive (ER^+^) and epithelial cells are often the sites of malignant transformation, MCF-7 cells represent a beneficial model for investigating the potential pro-estrogenic effects of alcohol [46, 47]. Furthermore, MCF-7 cells have been used previously to study alcohol-mediated effects. Xu *et al*. (2016) has demonstrated the effects of alcohol on MCF-7/MCF-ErbB2 cell and MMTV-Neu mouse models of the ErbB2-positive (ErbB2^+^) subtype of breast cancer [48]. In their study, alcohol increased cancer stem cell populations with colocalized activation of ErbB2, and promoted colon and lung metastases. Their molecular analysis of signaling pathways affected by alcohol stimulation, revealed the activation of MAPK p38, which is supported by *in vitro* work in our lab [11]. Our cellular model emphasizes the consequences of alcohol exposure on potentially cancer-initiating DNA damage in another breast cancer subtype, ER^+^/ErbB2-. Moreover, the isogenic knockdown of p53 in our MCF-7 cell line model is characteristic of breast cancers with p53 mutation/inactivation, which represents up to 70–80% of human breast cancers [29, 30].

Although a blood alcohol concentration (BAC) of 0.08% (17.4 mM) is the legal limit for driving in the United States and many other countries, much higher BACs can be reached following acute heavy alcohol consumption [49, 50]. As such, a BAC of 0.4% (87 mM) is typically lethal in humans. In our study using a range of physiologically relevant (0.1% and 0.2% v/v) and high/lethal concentrations (0.4% and 0.8% v/v) of alcohol, we first characterized alcohol-induced oxidative DNA damage in MCF-7 wild-type cells. As such, we showed that alcohol exposure (0.1–0.8% v/v) induced a dose-dependent increase in oxidative DNA damage, as indicated by quantification of 8-OHdG and staining for p-H2AX-positive foci, two known markers of oxidative DNA damage and DSBs, respectively (Fig 1) [40, 51]. As such, BRCA1 is a critical downstream protein of ATM and Chk2 that plays a central role in DNA repair by facilitating cellular responses to DNA damage and participating in major DNA repair mechanisms, including mismatch, nucleotide excision, base excision, and homologous recombination [52, 53]. Therefore, the activation of BRCA1 (Fig 2) implies that alcohol exposure may induce multiple types of DNA damage *in vitro*. Importantly, alcohol-induced DNA damage was associated with increased total and phosphorylated p53 (Fig 2). Concurrent induction of p-ATM, p-Chk2, and p-H2AX with p53 activation indicates that alcohol-induced p53 response involves the activation of the ATM/ATR-H2AX axis. Consistent with our *in vitro* alcohol-induced p53 activity, we found that acute alcohol exposure stimulates the activation of p53 and the DNA damage response network in premalignant mammary tissues of MMTV-ErbB2 transgenic mice (manuscript in preparation). Furthermore, we demonstrated that alcohol treatment stimulates the activation of the p53 promoter and the expression of p21 and Bax, two well-known p53 target genes, which may contribute to the cell cycle arrest in alcohol-treated cells *in vitro* (Fig 3). These results support the role of p53 as a checkpoint regulator that protects the cells from alcohol-induced genomic instability and transformation initiation. The conditions developed in these experiments also provide a foundation for studying p53-specific functions in the p53 knockdown cells.

Given the critical role of p53 in tumor suppression, whether/how p53 inactivation is involved in alcohol-associated breast carcinogenesis are important questions. However, the majority of studies attempting to address these questions used epidemiologic approaches with inconsistent results [27, 28, 54, 55]. In MCF-7 (p53 wild-type) cells with/without p53 knockdown, we characterized alcohol-regulated p53-targeted gene expression, cell cycle progression, and DNA damage. We demonstrated that inactivation of p53 by p53-specific siRNA significantly inhibits the expression of p53-targeted genes (i.e. p21 and Bax) and alleviated alcohol-induced cell cycle arrest (Fig 4). Data from these paired isogenic breast cancer cell lines provide unambiguous evidence showing the critical function of p53 in alcohol-induced cellular responses *in vitro*. Although we focused on p53-associated cell cycle arrest in this study, spontaneous apoptosis triggered by alcohol-induced DNA damage may be impaired in cells with p53 mutation/inactivation, as evidenced by the significant suppression of Bax and potentially other p53-regulated genes involved in apoptosis after alcohol exposure. The role of p53 in alcohol-associated spontaneous apoptosis as a protective response will be addressed in future studies. Our data from MCF-7/sip53 cells also show that alcohol induced a modest increase in p21, suggesting p53-independent pathways may regulate p21. Previously, it was reported that p21 and other cell cycle targets were induced in p53 mutants, indicating p53-independent cell cycle regulation [32–34]. Indeed, BRCA1 and p73 are found to directly stimulate p21 activity in the absence of functional p53; nevertheless, p53 appears to be the main regulator of cellular responses in our model [32–34, 38]. Consistent with the indicated role of p53 in the response to alcohol-induced DNA damage in our knockdown model, the stabilization of p53 via Nutlin-3 amplified alcohol-induced cell cycle arrest and the expression of downstream targets of p53 (Fig 6). Together, these results reiterate the central concept that p53 regulates cellular responses to DNA damage, including cell cycle arrest, DNA repair, and apoptosis. Moreover, as Nutlin-3 is an antagonist of Mdm2, an oncogene that promotes p53 degradation, use of Nutlin-3 not only provides additional support of our knockdown model, but also highlights the impact of functional p53 stabilization in alcohol-induced cellular responses.

Our results showed that alcohol increases protein levels of p-H2AX and numbers of p-H2AX foci in MCF-7 cells with p53 knockdown, as compared to the MCF-7 control cells (Fig 5). These data suggest that alcohol enhances DNA damage in cells with p53 mutation/inactivation. The mechanism underlying enhanced alcohol-induced DNA damage in MCF-7/sip53 cells may result from the decrease of anti-oxidant and DNA repair genes regulated by p53, such as SESN1 (an anti-oxidant gene) and DNA polymerase-β (a DNA repair gene involved in new strand synthesis) [56, 57]. In context with the notion that p53 mutations are associated with genomic instability, enhanced alcohol-induced DNA damage in these cells could form a vicious feedback loop that may promote malignant transformation.

In summary, we established an *in vitro* model system to study the specific role of p53 in alcohol-associated breast cancer risk and demonstrated that p53 inactivation significantly impairs cellular responses to alcohol-induced DNA damage. We also found that p53 inactivation results in enhanced DNA damage in alcohol-treated breast cancer cells, which forms a feedback loop and may promote tumor transformation. Taken together, our research underscores the importance of p53 in alcohol-associated breast cancer etiology and progression and lays the groundwork for future studies to explore the specific role of p53. Although our isogenic cell line model is an explicit example of p53 deletion, these paired cell lines can also be used to address alcohol-induced cellular responses in cells with p53 loss due to point mutations and functional inactivation. Future work will define the p53-mediated regulation of integral cell growth and survival mediators in response to alcohol stimulation and will evaluate the translational value of our *in vitro* findings in animal models. Promisingly, our current results are consistent with our *in vivo* findings (unpublished data). Our work will ultimately further our understanding of the fundamental processes that are implicated in alcohol-associated breast carcinogenesis and may potentially help develop personalized treatment strategies based on the p53 status of the tumor.
